# The deleterious effects of old social partners on *Drosophila* lifespan and stress resistance

**DOI:** 10.1038/s41514-022-00081-2

**Published:** 2022-03-18

**Authors:** Yu-Chiao Lin, MingYang Zhang, Sheng-Hao Wang, Chia-Wen Chieh, Pin-Yun Shen, Yi-Lin Chen, Yu-Chia Chang, Tsung-Han Kuo

**Affiliations:** 1 Department of Life Science, National Tsing Hua University, Hsinchu, Taiwan Republic of China; 2 Institute of Systems Neuroscience, National Tsing Hua University, Hsinchu, Taiwan Republic of China; 3 National Hsinchu Girls’ Senior High school, Hsinchu, Taiwan Republic of China

**Keywords:** Ageing, Sociology

## Abstract

Social interactions play important roles in the modulation of behavior, physiology, and, potentially, lifespan. Although longevity has been studied extensively in different model organisms, due to the complexity of social environments, the social modulation of aging remains poorly investigated. The present study used the fruit fly, *Drosophila melanogaster*, as a model to study lifespan and stress resistance under different social conditions. Our experiments first showed that social isolation increased fly lifespan, suggesting a potential deleterious effect of social companions. Furthermore, we exposed flies to different aged social partners and found that living with old animals significantly reduced lifespan and stress resistance in young animals. In contrast, living with young animals increased old animal lifespan, although the effects were less robust. Overall, our results suggest that while social interaction can influence fly health, specific social partners may have more pronounced effects than others. This study provides new evidence that different social environments have significant impacts on animal physiology and longevity.

## Introduction

Social interaction is an exchange of information or actions between two or more conspecifics that occurs in numerous species and plays an important role in regulating behavior and physiology^1–4^. The importance of social environments has been largely demonstrated by the harmful effects of social isolation in different species; these effects range from increased mortality in humans to the progression of a variety of diseases in model organisms^5–9^. The deleterious effects of social interaction, albeit less emphasized, have also been reported in different species^10,11^. Although the influence of social experiences and surroundings is a fascinating topic, due to extremely diverse and complicated social environments, research on the social modulation of health and physiology, especially aging, is very challenging and requires further extensive exploration.

With its small size and short lifespan, the fruit fly, *Drosophila melanogaster*, is a well-established model for the study of aging and physiology. While aging interventions involving the manipulation of diet or signaling pathways have been widely explored^12,13^, there is growing evidence showing positive and negative influences of social environments on fly aging. For example, although social isolation could reduce sleep^14^, increase food intake^15^, decrease mushroom body fibers^16^, and even induce cancer progression^17^, studies have shown a longer lifespan for flies under social isolation^18–20^. In addition, cohousing with normal young flies or flies with a longer lifespan can significantly increase the lifespan of short-lived mutants deficient in an antioxidant enzyme (*Sod*)^21^. Exposure to female pheromones can dramatically shorten the male lifespan^22^. Given the many social factors involved in interactions, various social partners with distinct genetic backgrounds, behaviors, or health conditions could potentially affect recipients differently.

Stress resistance was often investigated in fruit flies to reflect physiological conditions, and potentially longevity. Generally, animals with a longer lifespan tend to be more resistant to various forms of environmental stress. For example, in addition to lifespan changes, *Sod* mutants cohoused with health helpers were also more resistant to heat and oxidative stress^21^. Exposure to female pheromones not only shortened male lifespan but also decreased starvation resistance^22^. More importantly, several studies have successfully isolated long-lived mutants via manipulation of stress-responsive genes^23,24^, further indicating shared mechanisms between longevity and stress resistance.

In this report, we aimed to examine fly longevity under different social environments ranging from social isolation to the presence of different social companions. We first validated previous findings that flies under social isolation had a significantly longer lifespan than group-housed flies. Then, we showed that cohousing with old flies reduced lifespan in young animals. Multiple stress resistances were also decreased after living with old social partners. Together, our data suggested that social interactions, especially with old social partners, could have deleterious effects on fly health. This study therefore provides new evidence to demonstrate social impacts on animal longevity.

## Results

### Social isolation increased lifespan and stress resistance

**Fig. 1:**
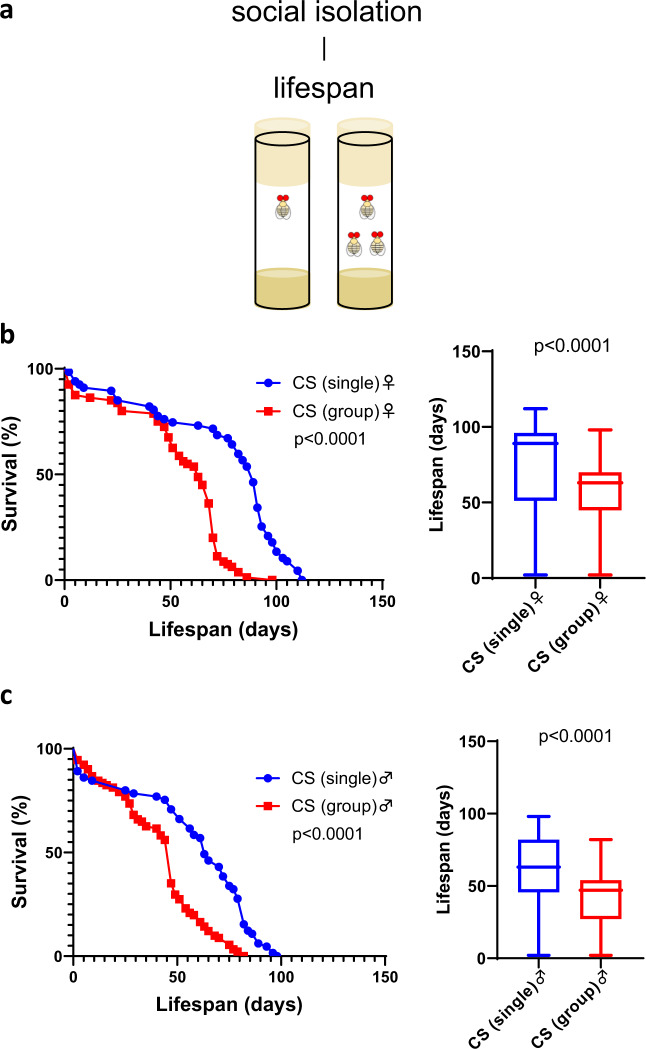
Social isolation extended fly lifespan. **a** Lifespan was compared between single-housed and group-housed flies. **b** Survival curves and survival days in females (*n* = 67 for single-housed and *n* = 80 for group-housed). **c** Survival curves and survival days in males (*n* = 65 for single-housed and *n* = 91 for group-housed). Survival curves were tested by the log-rank test. Survival days were tested by the Mann–Whitney *U* test. The boxplots show the minimum, 25th percentile, median, 75th percentile, and maximum values.

To investigate social influence, we first studied fly longevity under social isolation conditions by comparing the lifespans of single-housed and group-housed (30 flies in one vial) Canton-S flies (Fig. 1a). Cox regression suggested a significant difference according to housing condition (*P* < 0.001) and sex (*P* < 0.001). The interaction between sex and housing condition was close to but not statistically significant (*P* = 0.067). To investigate this further, we analyzed male and female lifespan separately and found that social isolation significantly increased lifespan in both sexes (Fig. 1b, c). These results are generally consistent with the previous studies^18–20^.

### Living with old social partners shortened lifespan

**Fig. 2:**
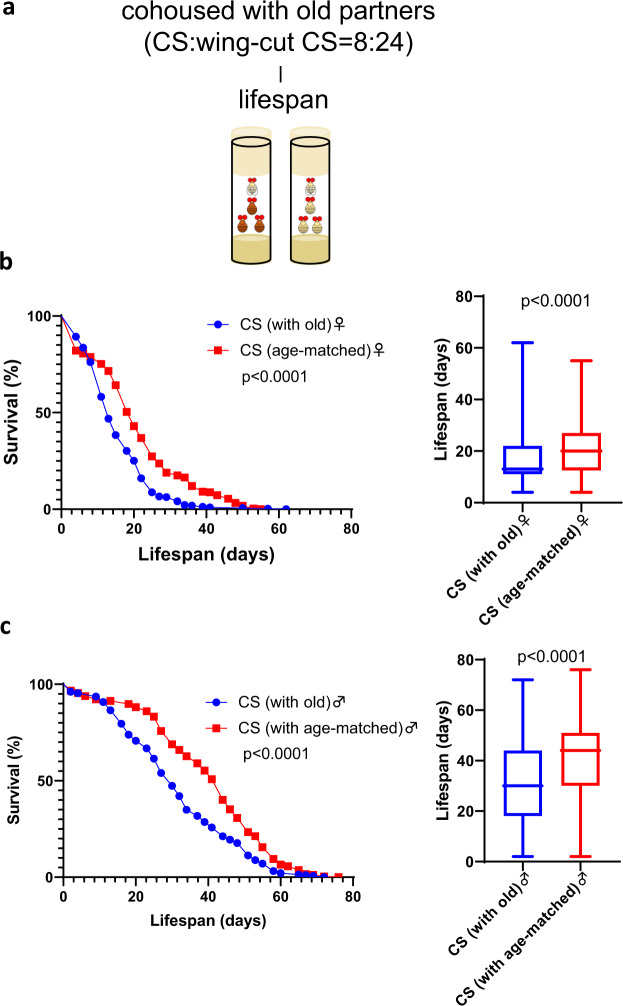
Cohousing with old donors shortened the lifespan of young Canton-S target flies. **a** Lifespan was examined in young Canton-S target flies cohoused with either old Canton-S or age-matched Canton-S donors at a 1:3 ratio. The donor flies were marked by wing clipping. **b** Survival curves and survival days in Canton-S target females (*n* = 318 for cohousing with old flies and *n* = 274 for cohousing with age-matched flies). **c** Survival curves and survival days in Canton-S target males (*n* = 283 for cohousing with old flies and *n* = 244 for cohousing with age-matched flies). Survival curves were tested by the log-rank test. Survival days were tested by the Mann–Whitney *U* test. The boxplots show the minimum, 25th percentile, median, 75th percentile and maximum values.

Since social interactions have strong deleterious effects, we next explored the social effects of different social partners. Specifically, we were interested in the social influence of flies of different ages, as age significantly changes animal behavior and physiology. We examined the lifespan of 1-day-old Canton-S target flies cohoused with 3 or 4 -week-old Canton-S donor flies marked by the clipped wing in a 1:3 ratio (Fig. 2a). The controls were 1-day-old Canton-S targets cohoused with aged-matched Canton-S donors marked by the clipped wing. According to Cox regression, the differences were statistically significant for sex (*P* < 0.001), social partner (*P* < 0.001) but not the interaction between these two factors (*P* = 0.257). Analyzing male and female lifespan separately showed that, for both sexes, cohousing with old donors significantly reduced the lifespan of targets (Fig. 2b, c).

Wing clipping may affect fly behaviors and social interaction. Although both our experimental and control target flies were cohoused with wing clipped donors, we decided to further verify this social effect by replacing Canton-S targets with mutants carrying visible markers (*w*^*1118*^ and *yw*) to avoid wing clipping. Consistent with the data from the Canton-S target, cohousing with old Canton-S donors reduced the lifespan of *w*^*1118*^ targets, although the difference in females was at the margin of statistical significance (Supplementary Fig. 1a–c). *yw* targets cohoused with old donors also exhibited a shorter lifespan than controls in both sexes (Supplementary Fig. 1d–f). Together, our results based on Canton-S, *w*^*1118*^, and *yw* suggested that living with old social partners could significantly reduce fly longevity. The results of mutant targets not only excluded the confounding effect of the clipped wings but also indicated the robustness of this phenomenon across genetic backgrounds.

### Living with old social partners decreased stress resistance

**Fig. 3:**
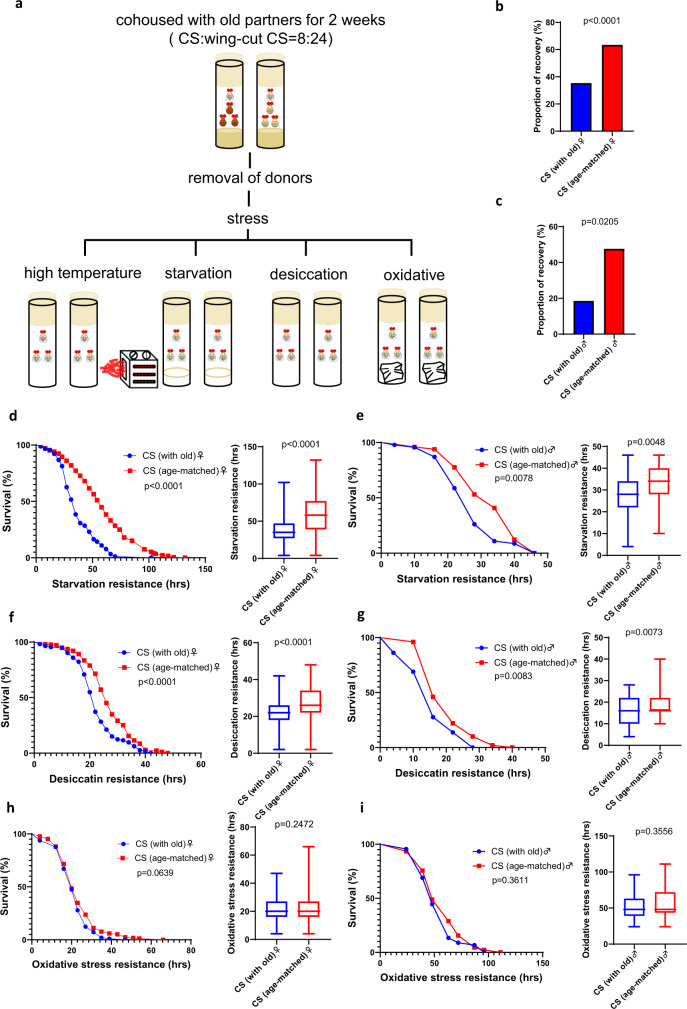
Cohousing with old donors decreased the stress resistance of Canton-S target flies. **a** Canton-S target males were cohoused with old Canton-S donors in a 1:3 ratio for 2 weeks. Then, the stress resistance of Canton-S targets was examined after the removal of donor flies. **b** The proportion of females that recovered from heat stress (*n* = 99 for cohousing with old flies and *n* = 104 for cohousing with age-matched flies). **c** The proportion of males that recovered from heat stress (*n* = 27 for cohousing with old flies and *n* = 42 for cohousing with age-matched flies). **d** Survival curves and survival hours in females under starvation stress (*n* = 91 for cohousing with old flies and *n* = 172 for cohousing with age-matched flies). **e** Survival curves and survival hours in males under starvation stress (*n* = 46 for cohousing with old flies and *n* = 49 for cohousing with age-matched flies). **f** Survival curves and survival hours in females under desiccation stress (*n* = 113 for cohousing with old flies and *n* = 175 for cohousing with age-matched flies). **g** Survival curves and survival hours in males under desiccation stress (*n* = 29 for cohousing with old flies and *n* = 50 for cohousing with age-matched flies). **h** Survival curves and survival hours in females under oxidative stress (*n* = 97 for cohousing with old flies and *n* = 169 for cohousing with age-matched flies). **i** Survival curves and survival hours in males under oxidative stress (*n* = 45 for cohousing with old flies and *n* = 45 for cohousing with age-matched flies). The percentages of recovery were tested by Fisher’s exact test. Survival curves were tested by the log-rank test. Survival hours were tested by the Mann–Whitney *U* test. The boxplots show the minimum, 25th percentile, median, 75th percentile, and maximum values. Error bars = SEM.

To investigate the physiological changes after cohousing, we next examined stress resistance in Canton-S target flies living with old social partners. Target flies were cohoused with old Canton-S flies for 2 weeks and then separated from donors for stress assays (Fig. 3a). In both sexes, flies previously cohoused with old donors showed significantly less resistance to heat, starvation, and desiccation stress (Fig. 3b–g). The reduced resistance to oxidative stress, however, was not significant (Fig. 3h, i). Starvation and oxidative resistance were also examined in *yw* targets cohoused with old Canton-S social partners (Supplementary Fig. 2a). Female flies showed reduced resistance to oxidative stress but not starvation stress (Supplementary Fig. 2b, c), while male flies showed reduced resistance to both stress resistance (Supplementary Fig. 2d, e).

### Living with old social partners reduced male courtship behaviors

**Fig. 4:**
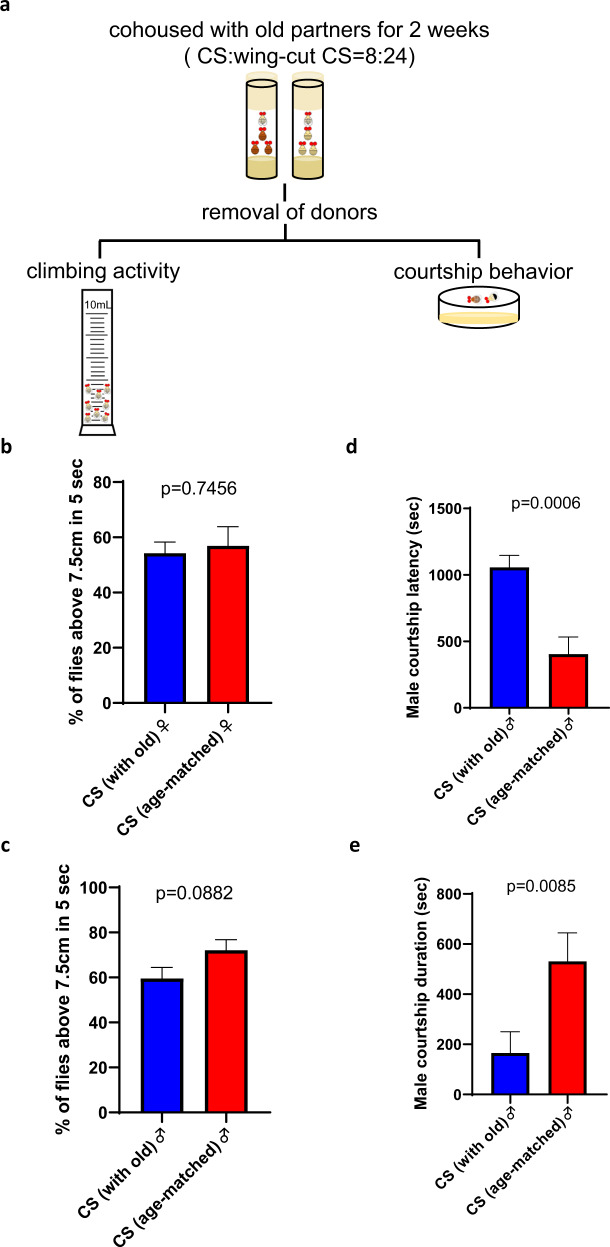
Cohousing with old donors decreased the courtship activity of Canton-S male flies. **a** Canton-S targets were cohoused with old Canton-S donors in a 1:3 ratio for 2 weeks. After the removal of donor flies, the climbing activity of Canton-S targets for both sexes and courtship activity for males were examined. **b** Percentage of female flies climbing over a 10 mL mark (*n* = 11 vials for cohousing with old flies and *n* = 11 vials for cohousing with age-matched flies). **c** Percentage of male flies climbing over 10 mL mark (*n* = 8 vials for cohousing with old and *n* = 7 vials for cohousing with age-matched flies). **d** Male courtship latency (*n* = 16 for cohousing with old flies and *n* = 15 for cohousing with age-matched flies). **e** Male courtship duration (*n* = 16 for cohousing with old flies and *n* = 15 for cohousing with age-matched flies). Climbing activity were tested with Student’s *t* test. Courtship activity was tested with the Mann–Whitney *U* test. Mean ± SEM.

To further explore the influence of old social partners, the activity of Canton-S target flies was examined by climbing assay after 2 weeks of cohousing (Fig. 4a). For both sexes, there was no significant difference in the percentage of flies climbing above the 10 ml mark (7.5 cm) in a 10 ml cylinder (Fig. 4b, c). On the other hand, we also examined male courtship activity and found that previous exposure to old donors largely increased courtship latency and reduced courtship duration (Fig. 4a, d, e), suggesting reduced courtship activity after living with old social partners. In sum, our results suggested that living with old social partners led to a shorter lifespan, less stress resistance, and lower male courtship activity.

### Young social partners do not consistently affect the lifespan of aged flies

**Fig. 5:**
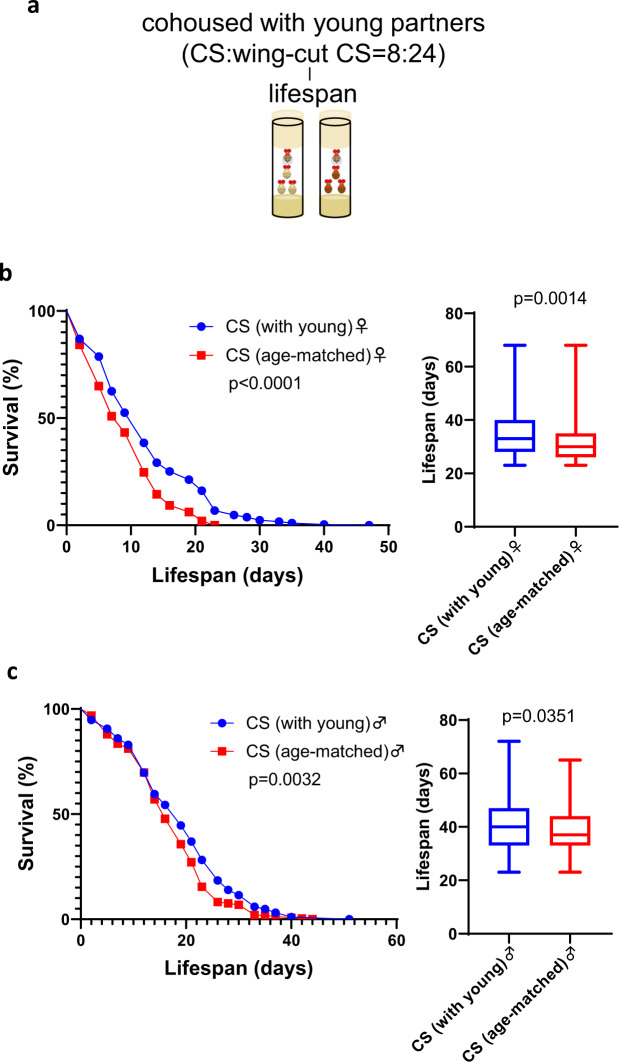
Cohousing with young donors extended the lifespan of old Canton-S target flies. **a** Lifespan was examined in old Canton-S target flies cohoused with either young Canton-S or age-matched Canton-S donors in a 1:3 ratio. The donor flies were marked by wing clipping. **b** Survival curves and survival days in Canton-S target females (*n* = 306 for cohousing with young flies and *n* = 291 for cohousing with age-matched flies). **c** Survival curves and survival days in Canton-S target males (*n* = 287 for cohousing with young flies and *n* = 291 for cohousing with age-matched flies). Survival curves were tested by the log-rank test. Survival days were tested by Mann–Whitney *U* test. The boxplots show the minimum, 25th percentile, median, 75th percentile, and maximum values.

The discovery of deleterious effects from old social partners led us to explore the impact of young partners. We hypothesized that exposure to young donor flies would extend the lifespan of old animals. To test this hypothesis, we examined the lifespans of 3-week-old Canton-S target flies cohoused with 1–3-day-old Canton-S donor flies (Fig. 5a). Cox regression suggested a significant difference in sex (*P* < 0.001), and social partner (*P* < 0.001) but not the interaction between sex and social partner (*P* = 0.303). Examination of males and females separately showed that, for both sexes, old flies cohoused with young donors lived significantly longer than flies cohoused with age-matched animals in both sexes (Fig. 5b, c). We also investigated this phenomenon in *w*^*1118*^ and *yw* targets. To enhance the social effect of youth, we replaced young donors every 2 weeks to maintain the youth of young donors. Unfortunately, although we observed lifespan extension in *w*^*1118*^ females cohoused with young Canton-S females (Supplementary Fig. 3a, b), compared to the control, males cohoused with young Canton-S males had reduced survival (Supplementary Fig. 3c). For *yw* as the target flies, we did not detect any significant change in either male or female lifespan (Supplementary Fig. 3d–f). Therefore, in contrast to the deleterious effect of old flies, we have no solid evidence to conclude that there is a beneficial influence of young social partners.

## Discussion

Our first experiment showed that single-housed flies have a significantly longer lifespan than group-housed flies. While the data seem to contradict general findings from human or rodent studies, the result was not unexpected. A few studies have already shown that social isolation can extend fly lifespan^18–20^. The disadvantage of group housing may be partially due to crowding stress or reduced environmental quality under high population density^25–27^. However, this lifespan shortening can also be observed in only pairs of flies^18^, suggesting harmful effects of social interactions. Therefore, even though social isolation has been shown to induce several undesirable effects in the fruit fly^14,17^, its impacts are probably less significant than the damage caused by social environments.

This study then investigated the influence of social partners and showed that living with older companions can be deleterious, not only for longevity but also for stress resistance. Since stress was applied after the removal of old social partners, the reductions in stress resistance were mainly caused by unfavorable health conditions rather than direct physical interactions. The 2-week cohousing before stress assays was chosen because the majority of flies were still alive for us to perform stress assays. At this stage, there is no significant effect of social partners on cumulative survival, suggesting that this social effect can be detected at an early age, long before the difference in lifespan. Unexpectedly, although we observed reduced courtship activity in males cohoused with old partners, the difference in climbing activity was not significant. These results implied that social partners could have a strong impact on courtship motivation but maybe not general locomotion activity. It is worth noting that both stress resistance and courtship activity assays were conducted right after CO_2_ anesthesia, which could potentially affect physiology and behavioral performances^28,29^. Other approaches, such as cold anesthesia, should be considered for similar experiments in future research.

Our data also implied a beneficial influence of young companions on old flies, although the magnitudes of the change in average lifespan (female 6.9%; male 4.9%) seemed to be much smaller than the influence of old companions on young flies (female 26.2%; male 22.3%). These results were overall consistent with a recent report suggesting that perception of young flies could extend old fly lifespan^30^. The effects of old partners on young individuals were also implied in this study. Although their and our experiments focused on different outcomes (old partners’ effects vs. young partners’ effects), both studies attempted trying to address the question of whether fly lifespan can be modulated to reflect the lifespan of their social partners of different ages. There were several key differences in the experimental designs of these two studies. For example, the population density in their experiments (20 flies per vial) was lower than ours (32 flies per vial), which might be the reason for the stronger young effect in their study. Virgin flies were tested in their studies while we focused on mated animals. Most importantly, because wing clipping could cause substantial damage and affect fly behaviors, physiology, and potentially longevity, we distinguished donor and target flies by removing donors’ wings to keep intact target flies. In their experiments, however, the wings of target flies were being removed. Nevertheless, despite the differences in the experimental designs, both studies suggested that fly longevity can be influenced by young or old social partners. Unfortunately, our experiments based on *w*^*1118*^ and *yw* did not show consistent effects from young partners. Since targets and donors in these mutant experiments were different strains, the inconsistency can be caused by the confounding effect of genetic background. Alternatively, because new donors were supplemented in *w*^*1118*^ and *yw* experiments every two weeks to enhance the youth environment, failure to detect young social effects might be due to the loss of familiarity of partners, which in lifespan regulation remains to be explored.

How old flies shorten lifespan and reduce stress resistance in young flies remains to be further explored. The presence of old flies could introduce several changes in the environment to shorten the target lifespan. Density, for example, is known to exert a critical effect on fly longevity^25,26^. However, we reasoned that density change is less likely to be the cause of lifespan shortening in our study because old flies with a higher death rate would lead to a lower density, which should increase rather than decrease the target lifespan. In contrast, the study by Cho et al. implied that lifespan extension by young partners was caused by the perception of cuticular pheromones^30^, which was more likely to be a mechanism involved in lifespan shortening by old partners. Cohousing with old flies would certainly increase the possibility of encountering dead flies, which has been reported to decrease lifespan as well^31^. In addition, a recent study showed that social interactions with older partners can alter male microbiomes^32^, which could be functionally linked to physiology and longevity. For example, a greater pathogen load on old or dead flies could potentially decrease the fly’s lifespan. Although all food was supplemented with antibiotics, old flies with weaker immunity could still carry more infectious pathogens, which are transferred to target flies during social interactions. One or all of these factors could be possible mechanisms to reduce longevity. While the present study only focuses on stress resistance and activity, future studies investigating other physiological or molecular changes would help us to examine these potential factors and identify the mechanisms underlying this lifespan modulation by old social partners.

Our discovery of the social influences on fly longevity implied a nonautonomous effect on lifespan regulation. Since most aging studies in fruit flies were conducted in group housing environments, our findings suggested that the lifespan changes in previous studies may partially be caused by social interactions in addition to experimental manipulations. For example, lifespan extension under diet restriction was not only caused by food manipulation but also by interactions with healthy social partners. This could also explain why single-housed fly without old social partners can live longer. We therefore expected that the impacts of lifespan interventions should be significantly lower without social interaction, at least in fruit flies. Testing lifespan manipulations under social isolation conditions would help us to address this hypothesis in the future.

In summary, our study provides new evidence that social environments significantly regulate animal physiology and lifespan. Specifically, we showed that social interactions can be deleterious, at least in the fruit fly. The positive or negative effect of social influence might largely depend on the life history of the experimental species. Unlike humans, flies spend more time alone in the wild and interact with each other occasionally during feeding or mating. Social interactions under laboratory housing conditions are thus much more intense than interactions in wild environments and more likely to have damaging impacts. In fact, although most studies, especially those in humans, preferred to emphasize the beneficial effects of social interactions, some types of social contacts could be harmful, such as social conflicts or social stress. Our understanding of social influences on animal or human health or aging is still in a very early stage. The fruit fly, which has a simple social life and short lifespan, presents a good model for the study of the social modulation of aging. Future research focusing on the mechanisms underlying this modulation would provide important information related to the regulation of aging by social environments.

## Methods

### Fly stocks and environmental details

The laboratory stocks Canton-S (CS), *w*^*1118*^, and *yellow-white* (*yw*) were obtained from Fly Core in Taiwan. The fruit fly is currently not subject to any ethical restrictions in Taiwan. Flies were maintained on standard white food composed of yeast, corn powder, agar, antibiotics, and preservatives at 25 °C and 60% RH humidity under 12/12 h day–night cycle. The detailed composition of fly food is listed in Supplemental Table 1.

### Manipulation of social environments

For lifespan, stress assays, and climbing assay, flies that emerged in bottles were transferred within 3 days to new bottles where they were allowed to mate freely for 24 h. For the courtship assay, newly emerged flies were collected within 8 h. Flies were then sexed under carbon dioxide anesthesia and placed in vials according to the cohousing conditions.

#### Social isolation

Canton-S flies were reared individually in each vial for single-housed conditions. The group-housed flies were started with a group of 30 flies in one vial.

#### Cohousing with old donors

Three- to four-week-old Canton-S flies were used as donors to be cohoused with young target flies. For Canton-S target flies, 8 targets and 24 donors, which were marked by wing clipping, were housed in one vial. For *w*^*1118*^ target flies, 8 targets and 24 donors were housed in one vial. For *yw* target flies, 10 targets and 20 donors were housed in one vial.

#### Cohousing with young donors

One to three-day-old Canton-S flies were used as donors to be cohoused with 3–4-week-old target flies. For Canton-S target flies, 8 targets and 24 donors, which were marked by wing clipping, were housed in one vial. For *w*^*1118*^ target flies, 8 targets and 24 donors were housed in one vial. For *yw* target flies, 10 targets and 20 donors were housed in one vial. In the *w*^*1118*^ and *yw* experiments, 1–3-day-old donors were supplemented every two weeks to maintain the youth of young donors.

### Lifespan and stress assays

For lifespan experiments, flies were transferred to new vials, and death counts were recorded every 2–3 days. For stress resistance, vials were transferred every 2–3 days for 2 weeks. Then, target flies were separated from donors under carbon dioxide anesthesia for stress assays.

#### Oxidative experiment

The oxidative resistance assay was based on a previous study^21^. Eight to ten target flies were placed in each vial containing a small piece of Kimwipe tissue and 340 μL of hydrogen peroxide solution (5% sugar and 3% hydrogen peroxide). Another 70 μL of hydrogen peroxide solution was supplemented every afternoon. Death counts were recorded every 4–6 h.

#### Starvation experiment

The starvation resistance assay was based on a previous study^22^. Eight to ten target flies were placed in each vial containing 5 mL of 5% agar as a water source. Death counts were recorded every 4–6 h.

#### Desiccation experiment

The desiccation resistance assay was modified from previous studies^33,34^. Eight to ten target flies were placed in each empty vial. Due to the short lifespan under desiccation, death counts were recorded every 2–4 h.

#### Heat stress experiment

The heat stress resistance was modified from a previous study^21^. Thirty target flies in each empty vial were moved to a 37 °C incubator for 80 min (for males) and 120 min (for females). The incubation time was different between the two sexes because females are generally more resistant than males. After recovery at room temperature, the number of active flies was recorded.

### Climbing assay

The climbing assay was used to measure the activity ability^35^. Eight to ten target flies were transferred to a 10-mL cylinder without anesthesia. The cylinder was then rapidly knocked on the desk three times to drop down all flies to the bottom. The climbing ability of flies was recorded by video for data analysis. The percentage of flies climbing above 7.5 cm (10 mL mark) in 5 s was recorded.

### Courtship behavior assay

Virgin males and females were collected within 8 h of emergence for courtship behavioral experiments. For the courtship arena, each well of a 24-well plate was filled with standard white food. One target male that had been cohoused for 2 weeks and a 7-day-old virgin female were placed on either side of a well, which was divided by a clapboard. After 40 min of habituation, the clapboard was removed, and the total male courtship duration and courtship latency were recorded for 20 min.

### Statistical analysis

Statistical analyses were performed by R software and GraphPad Prism. For lifespan, interactions between sex and housing condition were examined by Cox regression. Survival curves for lifespan, starvation, oxidation, and desiccation stress resistance assays were tested by the log-rank (Mantel Cox) test. Survival days were tested by the two-tailed Mann–Whitney *U* test. The boxplots show the median values, with the box passing through the 25th–75th percentiles. The top and bottom lines show the maximum and minimum lifespan values. Fisher’s exact test was used for the heat stress experiment. Climbing activity were tested with two-tailed Student’s *t* test. Courtship activity was tested with the two-tailed Mann–Whitney *U* test.

## Supplementary information

Supplementary information

## Data Availability

All data are available in the paper or the supplementary materials; raw data are available upon request.
