# Supplementary material for: The deleterious effects of old social partners on Drosophila lifespan and stress resistance
Source: NPJ Aging. 2022 Mar 18;8(1):1. doi: 10.1038/s41514-022-00081-2 (PMC9158773; doi:10.1038/s41514-022-00081-2)
Supplement: Supplementary file 1 — Supplementary information [file 41514_2022_81_MOESM1_ESM.pdf]

Supplementary Table S1 Composition of *Drosophila* growth medium.

Ingredient	Volume	Ingredient	Volume
**Main contents**		**Antibiotic mixture**	
ddH_2O	1 L	Ampicillin	38.8 mg/L
Maltodextrin	57.45 g/L	Chloramphenicol	38.8 mg/L
Corn starch	69.82 g/L	95% ethanol	4.73 ml/L
Sugar	28.85 g/L	ddH_2O	4.73 ml/L
saccharomyces cerevisiae	29.33 g/L	Calcium chloride dihydrate	664.2 mg/L
Agar	8.48 g/L	**Preservative**	
Potassium sodium tartrate	8 g/L	95% ethanol	11.39 ml/L
		Methyl p-hydroxybenzoate	2.67 g/L

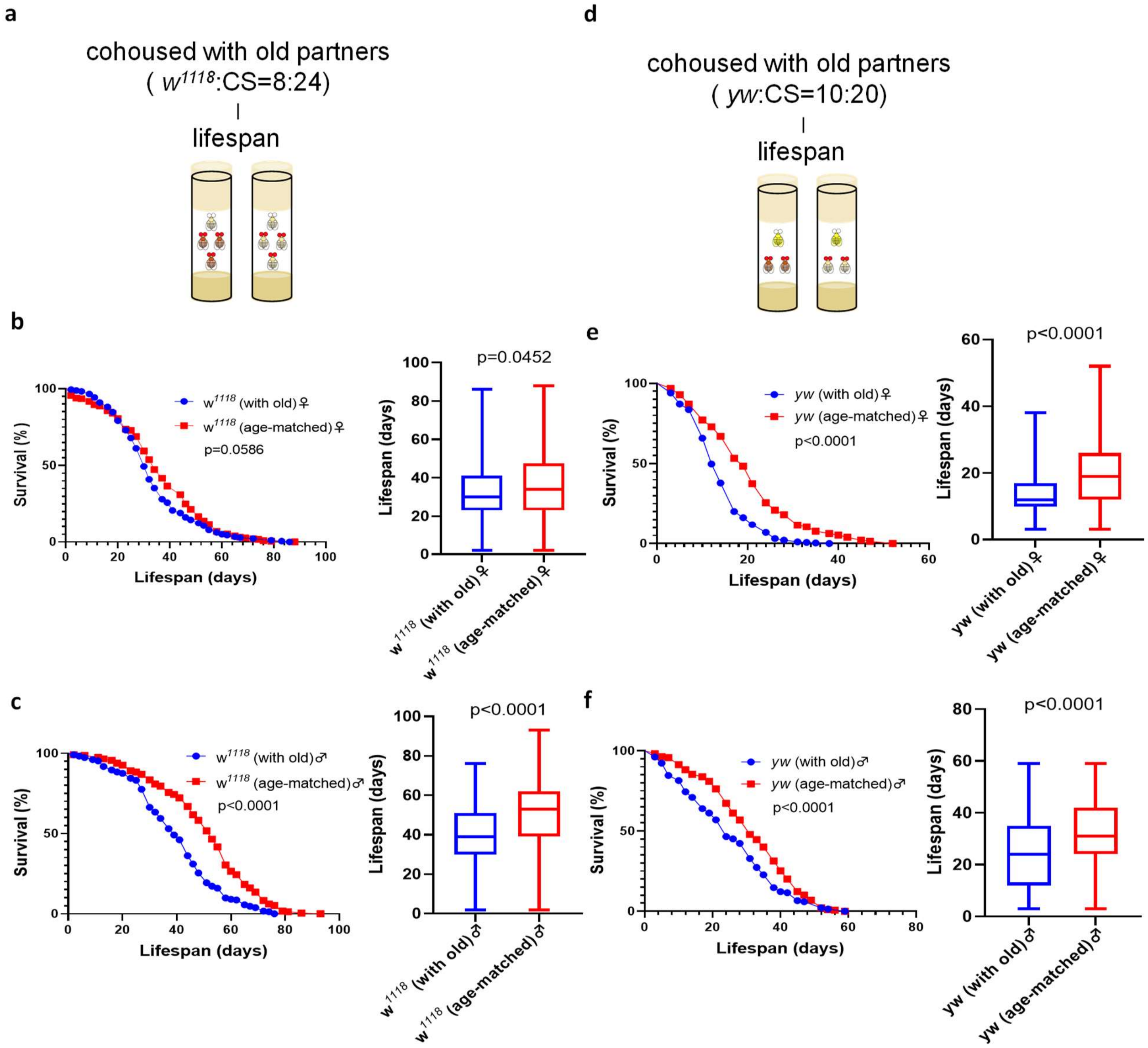
a
cohoused with old partners
(w1118:CS=8:24)
lifespan
b
w1118 (with old)♀
w1118 (age-matched)♀
p=0.0586
Survival (%)
Lifespan (days)
p=0.0452
Lifespan (days)
w1118 (with old)♀
w1118 (age-matched)♀
c
w1118 (with old)♂
w1118 (age-matched)♂
p<0.0001
Survival (%)
Lifespan (days)
p<0.0001
Lifespan (days)
w1118 (with old)♂
w1118 (age-matched)♂
d
cohoused with old partners
(yw:CS=10:20)
lifespan
e
yw (with old)♀
yw (age-matched)♀
p<0.0001
Survival (%)
Lifespan (days)
p<0.0001
Lifespan (days)
yw (with old)♀
yw (age-matched)♀
f
yw (with old)♂
yw (age-matched)♂
p<0.0001
Survival (%)
Lifespan (days)
p<0.0001
Lifespan (days)
yw (with old)♂
yw (age-matched)♂

Supplementary Figure 1 Cohousing with old Canton-S donors shortened the lifespan of young *w*^{1118} and *yw* target flies. a Lifespan was examined in *w*^{1118} target flies cohoused with either old Canton-S or age-matched Canton-S donors at a 1:3 ratio. **b** Survival curves and survival days in *w*^{1118} target females (n = 178 for cohousing with old flies and n = 232 for cohousing with age-matched flies). **c** Survival curves and survival days in *w*^{1118} target males (n = 232 for cohousing with old flies and n = 230 for cohousing with age-matched flies). **d** Lifespan was examined in *yw* target flies cohoused with either old Canton-S or age-matched Canton-S donors in a 1:2 ratio. **e** Survival curves and survival days in *yw* target females (n = 287 for cohousing with old flies and n = 285 for cohousing with age-matched flies). **f** Survival curves and survival days in *yw* target males (n = 286 for cohousing with old flies and n = 278 for cohousing with age-matched flies). Survival curves were tested by the log-rank test. Survival days were tested by the Mann–Whitney U test. The boxplots show the minimum, 25th percentile, median, 75th percentile and maximum values.

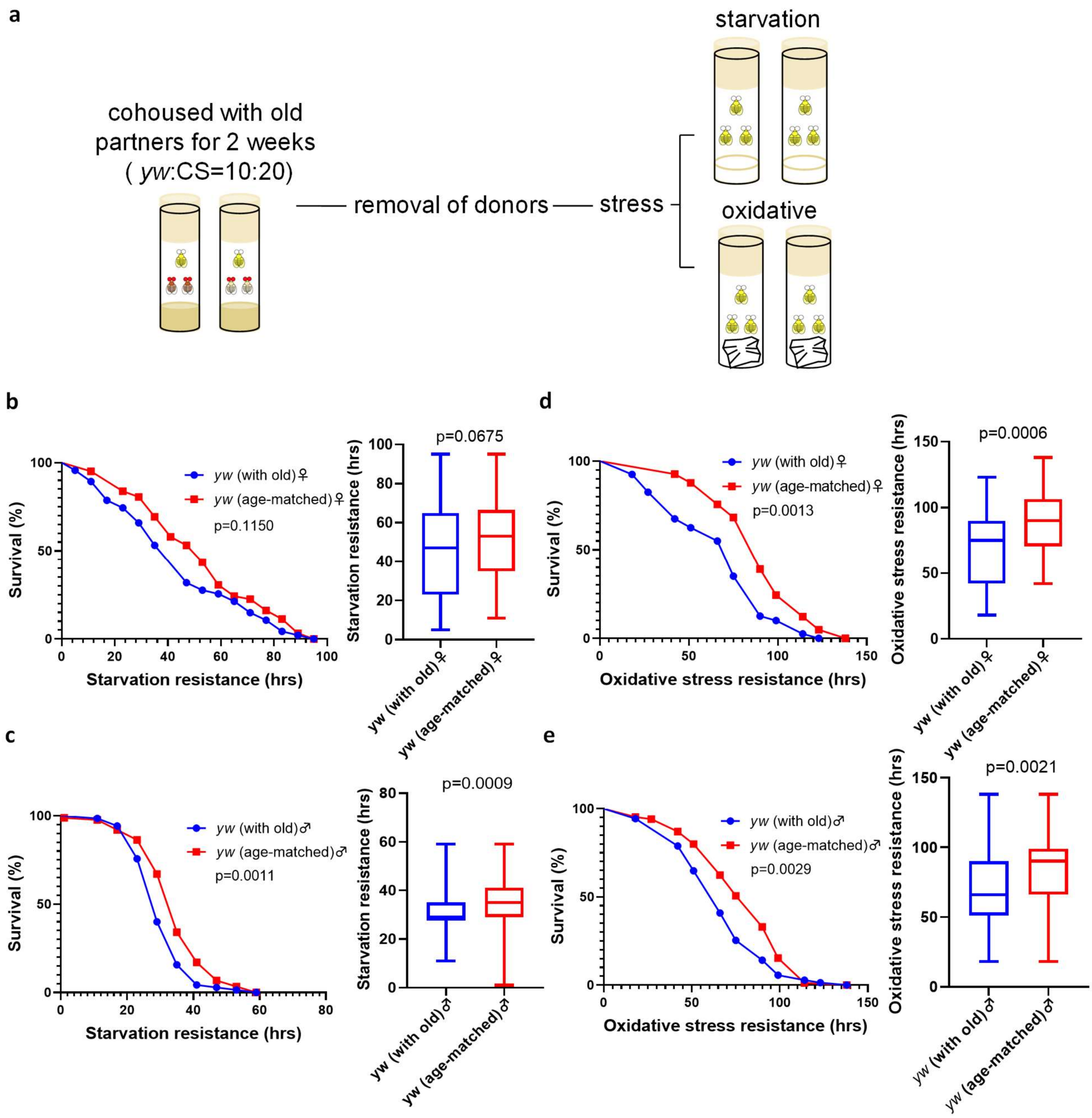
a
starvation
cohoused with old
partners for 2 weeks
(yw:CS=10:20)
removal of donors
stress
oxidative
b
yw (with old)♀
yw (age-matched)♀
p=0.1150
Survival (%)
Starvation resistance (hrs)
p=0.0675
Starvation resistance (hrs)
yw (with old)♀
yw (age-matched)♀
c
yw (with old)♂
yw (age-matched)♂
p=0.0011
Survival (%)
Starvation resistance (hrs)
p=0.0009
Starvation resistance (hrs)
yw (with old)♂
yw (age-matched)♂
d
yw (with old)♀
yw (age-matched)♀
p=0.0013
Survival (%)
Oxidative stress resistance (hrs)
p=0.0006
Oxidative stress resistance (hrs)
yw (with old)♀
yw (age-matched)♀
e
yw (with old)♂
yw (age-matched)♂
p=0.0029
Survival (%)
Oxidative stress resistance (hrs)
p=0.0021
Oxidative stress resistance (hrs)
yw (with old)♂
yw (age-matched)♂

Supplementary Figure 2 Cohousing with old donors decreased the stress resistance of *yw* target flies. a *yw* target flies were cohoused with old Canton-S donors in a 1:2 ratio for two weeks. Then, the stress resistance of *yw* target flies was examined after the removal of donor flies. **b** Survival curves and survival hours in females under starvation stress (n = 47 for cohousing with old flies and n = 62 for cohousing with age-matched). **c** Survival curves and survival hours in males under starvation stress (n = 70 for cohousing with old flies and n = 88 for cohousing with age-matched flies). **d** Survival curves and survival hours in females under oxidative stress (n = 40 for cohousing with old flies and n = 41 for cohousing with age-matched flies). **e** Survival curves and survival hours in males under oxidative stress (n = 71 for cohousing with old flies and n = 85 for cohousing with age-matched flies). Survival curves were tested by the log-rank test. Survival hours were tested by the Mann–Whitney U test. The boxplots show the minimum, 25th percentile, median, 75th percentile and maximum values.

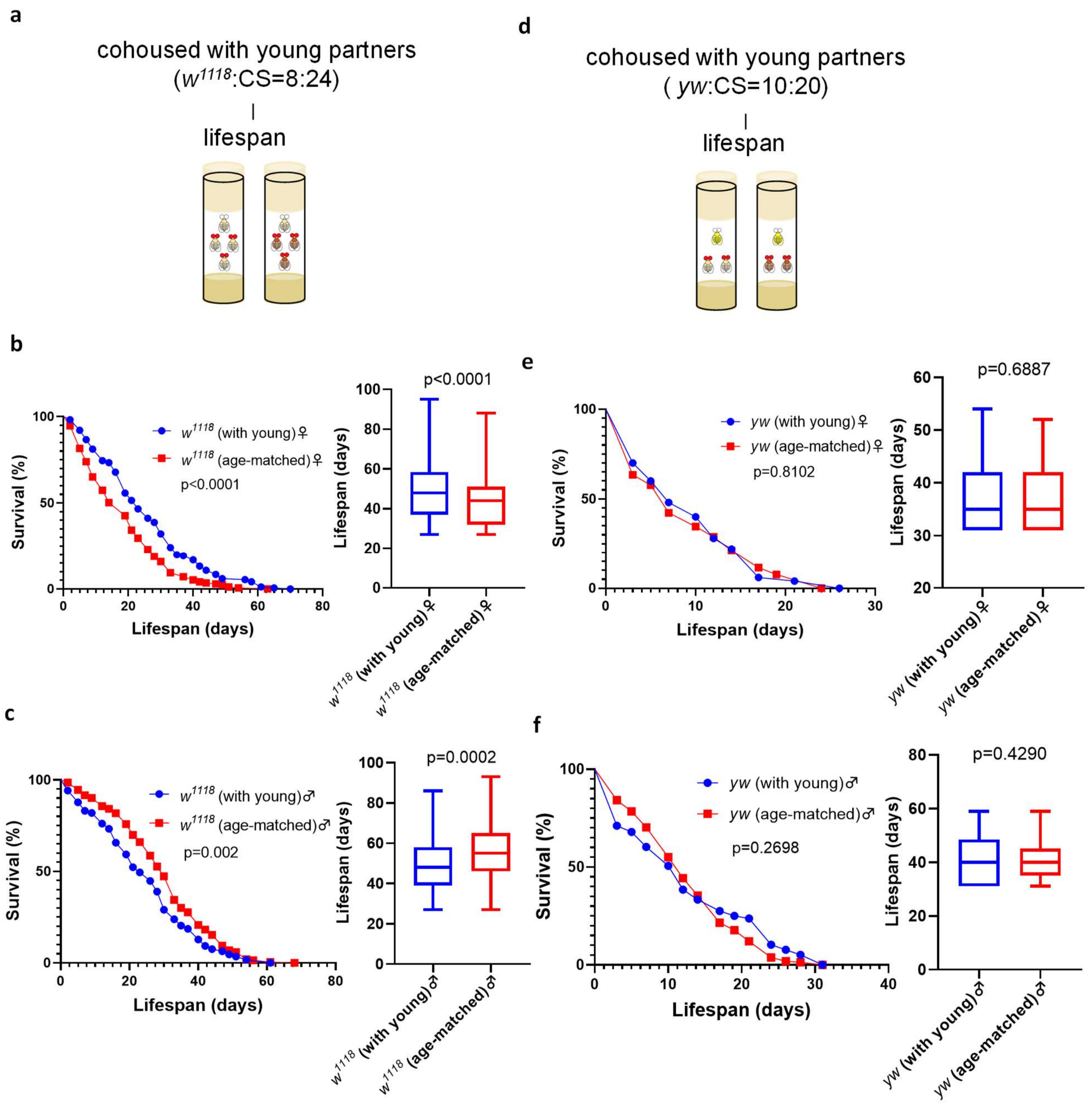
a
cohoused with young partners
(w1118:CS=8:24)
lifespan
b
Survival (%)
Lifespan (days)
w1118 (with young)♀
w1118 (age-matched)♀
p<0.0001
Lifespan (days)
p<0.0001
w1118 (with young)♀
w1118 (age-matched)♀
c
Survival (%)
Lifespan (days)
w1118 (with young)♂
w1118 (age-matched)♂
p=0.002
Lifespan (days)
p=0.0002
w1118 (with young)♂
w1118 (age-matched)♂
d
cohoused with young partners
(yw:CS=10:20)
lifespan
e
Survival (%)
Lifespan (days)
yw (with young)♀
yw (age-matched)♀
p=0.8102
Lifespan (days)
p=0.6887
yw (with young)♀
yw (age-matched)♀
f
Survival (%)
Lifespan (days)
yw (with young)♂
yw (age-matched)♂
p=0.2698
Lifespan (days)
p=0.4290
yw (with young)♂
yw (age-matched)♂

Supplementary Figure 3 Cohousing with young donors has no consistent effect on old target flies. **a** Lifespan was examined in old w^{1118} target flies cohoused with either young Canton-S or age-matched Canton-S donors in a 1:3 ratio. **b** Survival curves and survival days in w^{1118} target females (n = 165 for cohousing with young flies and n = 169 for cohousing with age-matched flies). **c** Survival curves and survival days in w^{1118} target males (n = 172 for cohousing with young flies and n = 203 for cohousing with age-matched flies). **d** Lifespan was examined in old *yw* target flies cohoused with either young Canton-S or age-matched Canton-S donors in a 1:2 ratio. **e** Survival curves and survival days in *yw* target females (n = 50 for cohousing with young flies and n = 52 for cohousing with age-matched flies). **f** Survival curves and survival days in *yw* target males (n = 156 for cohousing with young flies and n = 158 for cohousing with age-matched flies). Survival curves were tested by the log-rank test. Survival days were tested by the Mann–Whitney U test. The boxplots show the minimum, 25th percentile, median, 75th percentile and maximum values.
